# Curriculum Innovations: How Real Is Real Enough? A Pilot Study Comparing Standardized Patients vs Manikin Simulators in a Neurologic Emergencies Training Course

**DOI:** 10.1212/NE9.0000000000200004

**Published:** 2022-09-26

**Authors:** Catherine S.W. Albin, Emil Petrusa, James A. Gordon, Deepa Malaiyandi, Sahar F. Zafar

**Affiliations:** From the Department of Neurology (C.A.), Emory University School of Medicine, Atlanta, GA; Departments of Surgery (E.P.), and Emergency Medicine (J.A.G.), Massachusetts General Hospital, Harvard Medical School, Boston; MGH Learning Laboratory (E.P, J.A.G), Massachusetts General Hospital, Boston; Department of Neurology (D.M.), University of Toledo, OH; and Department of Neurology (S.F.Z.), Massachusetts General Hospital, Harvard Medical School, Boston.

## Abstract

**Background and Objectives:**

Simulation training has been increasingly adopted in neurology as an engaging way to promote active learning in a safe environment while offering a reproducible platform for real-time feedback. However, despite the increase in simulation programs, there remains uncertainty about which type of high-fidelity platform would best promote trainee knowledge and confidence acquisition. The objective of this study is to investigate whether increases in resident knowledge and confidence differ when a simulation course for acute neurology emergencies uses a standardized patient vs a manikin-video format. We also investigated trainees' management deviations from the treatment guidelines.

**Methods:**

Over 5 sessions, 20 junior neurology residents participated in a simulation training course in which they managed 3 neurologic emergencies: right middle cerebral artery stroke, status epilepticus, and pontine hemorrhage causing coma. Residents in the standardized patient group interacted with a live actor for the cases in which the patient was conscious. Residents in the manikin-video group interacted with a manikin for all 3 cases. Before and after the course, residents completed a 40-question multiple-choice test and a survey about their self-perceived confidence in handling 15 neurologic emergencies. To create an element of internal validity, 9 items were represented in the course curriculum and 6 were not. During the simulation, a detailed behavior checklist was used to assess decision-making and guideline adherence. All residents answered items about the educational quality of the simulation sessions.

**Results:**

Residents had significantly higher scores on the knowledge assessment after the training session (pre: 49% vs post: 72%, *p* < 0.001, effect size 91%). There was no statistically significant difference between the 2 groups—each increasing 23% (*p* = 0.977). Regardless of group assignment, the median self-reported confidence score improved by 1 point on a Likert scale across the topics taught in the course. The behavior checklist demonstrated significant variations in treatment practices and provided targeted areas for feedback and teaching.

**Discussion:**

This pilot study suggests that trainees' knowledge and confidence in the management of neurologic emergencies increase after simulated encounters, regardless of whether a live actor or manikin simulation platforms is used. The use of a behavior checklist uncovered important variations in guideline adherence among novice physicians.

Neurology trainees commonly serve as first-line responding clinicians for neurologic emergencies.^1,2^ Many neurology residency programs report that a large proportion of their residents' knowledge of neurocritical care principles is obtained through traditional lecture-only didactic series and self-directed learning.^1^ Unlike traditional learning, simulation offers trainees the opportunity to participate in deliberate, standardized practice, as well as receive timely, directed feedback on management without the risk of harm to patients.

There is mounting evidence that simulation training can improve performance in acute ischemic stroke,^3,4^ increase trainee knowledge and comfort with brain death testing,^5-8^ and improve adherence (or demonstrate nonadherence) to the epilepsy guidelines for status epilepticus.^9,10^ Yet, despite a growing body of evidence that supports simulation training, there remains uncertainty about how best to create an authentic simulated experience for neurology trainees, given that neurologic findings may be difficult to replicate with a manikin.^9,10^ Pioneering studies have used manikin-based curriculum,^4,6,7,9,10^ standardized patient-based curriculum,^3^ or a hybrid of the 2.^5,8^ However, within neurology, it is not known whether knowledge and/or confidence differentially increase when the simulated environment uses a standardized patient or a manikin simulator to portray a conscious patient.

The primary aim of this study was to compare the effect of participating in one of the 2 high-fidelity simulation courses on resident knowledge and confidence. The courses were the same except that in the one format, a manikin was used, regardless of patient consciousness, and in the other, a standardized patient acted in the case if the patient was conscious. Our secondary aim was to investigate the areas of trainee deviation from the current treatment guidelines in the diagnosis and management of acute ischemic stroke, status epilepticus, and acute coma with elevated intracranial pressure (ICP).

## Methods

### Study Design, Enrollment, and Randomization

The protocol was submitted and approved by The Massachusetts General Hospital Institutional Review Board. This prospective, nonblinded study was performed at the Massachusetts General Hospital's simulation center during July and August 2018. All residents signed written consent to participate.

Eighteen postgraduate year (PGY)-2 adult neurology residents and 2 PGY-3 child neurology residents (n = 20 participants) participated in the simulation curriculum as part of a new training innovation. Residents were assigned to one of the 5 dates according to their clinical schedules because the simulations required a morning free of clinical responsibilities. Residents (n = 10) who were assigned to the first, second, or fifth training date were assigned to the standardized patient group (group SP) and completed a curriculum in which there was a live actor for cases with a conscious patient (the acute ischemic stroke and seizure cases). Residents (n = 10) who were assigned to the third or fourth trainee date were assigned to the manikin with video supplement group (group MV). These residents interacted with a manikin for the entire course. Regardless of platform assignment, all residents completed the same 3 cases: right middle cerebral artery (MCA) acute ischemic stroke, status epilepticus, and pontine hemorrhage causing coma.

During the simulation course, residents worked as a group (3–5 residents per team) and were encouraged to appoint a different team leader for each case. The team leader was instructed to assign roles to other team members; roles included gathering history, examining the patient, reviewing vital signs, and administering medications.

### Cases

A neurology-boarded medical simulation fellow (C.A.) and board-certified neurointensivist (S.F.Z.) constructed the 3 cases for the curriculum based on commonly encountered diagnoses that have Neurocritical Care Society–endorsed protocols to guide best practice^13-15^—ischemic stroke, coma/intracerebral hemorrhage (ICH), and status epilepticus. In addition, these cases were selected for their relevance to specific Accreditation Council for Graduate Medical Education milestones for neurology trainees.^17^

Table 1 summarizes the major clinical objectives for each case. Objectives were based on the Neurocritical Care Society–endorsed guidelines^13-15^ and were determined by a neurologist (C.A.) and neurointensivist (S.F.Z.). A detailed behavior checklist for each case is available in eTable 1 (links.lww.com/NE9/A3). All 3 complete cases are available in the supplemental material (eCase ischemic stroke, links.lww.com/NE9/A0; eCase intraparenchymal hemorrhage, links.lww.com/NE9/A1; and eCase status epilepticus, links.lww.com/NE9/A2).

**Table 1:** Major Clinical Objectives for Each Case

Case 1 (acute ischemic right MCA stroke case)	Case 2 (status epilepticus case)	Case 3 (coma and pontine hemorrhage case)
Recognize hemiplegic weakness, neglect, and gaze deviation as consistent with an acute ischemic stroke Calculate an NIHSS Screen for contraindications to tPA Administer tPA with correct dosing once BP is appropriately controlled Identify criteria to involve the endovascular team Recognize the differential for acute worsening in neurologic examination after tPA: hemorrhage vs blood pressure–dependent examination	Formulate a differential diagnosis and an appropriate laboratory workup for subacute neurologic deficits Respond to acute onset seizure Recognize status epilepticus and implement guideline-recommended treatment for status epilepticus. Review indications for intubation in patients with status epilepticus Formulate a differential diagnosis of first-time seizure Recognize significant, symptomatic vasogenic edema	Identify features for coma caused by a structural etiology Perform a coma examination (mental status, cranial nerves testing, noxious stimulation to all extremities) Recognize the effect of sedation on the neurologic examination Order appropriate neuroimaging: head CT and CTA head and neck Institute management of ICH: BP control and reverse coagulopathy Identify radiographic features of hydrocephalus Consult neurosurgery

Abbreviations: BP = blood pressure; CTA = CT angiogram; ED = emergency department; ICH = intracerebral hemorrhage; ICP = intracranial pressure; MCA = middle cerebral artery; NIHSS = NIH Stroke Score; NSGY = neurosurgery; tPA = tissue plasminogen activator.

#### Case 1

The first case was a patient with a right MCA stroke who met criteria for tissue plasminogen activator (tPA) administration and endovascular thrombectomy. The case required trainees to accurately identify an acute stroke, perform a NIH Stroke Scale (NIHSS), order urgent imaging, screen and consent for tPA, and consider endovascular intervention. After tPA administration, the patient clinically deteriorated because of an acute drop in blood pressure. This change required trainees to consider the differential for patient deterioration after tPA administration.

#### Case 2

The second case demonstrated a patient with a subacute mental status change secondary to a large and previously undiagnosed frontal tumor. While residents considered the cause of these behavioral changes, the patient had a prolonged seizure, which did not terminate with the first round of benzodiazepine treatment and met criteria for status epilepticus.^15^ Trainees were required to demonstrate appropriate airway management, benzodiazepine dosing, and choice of antiseizure medication. When obtained, the CT scan revealed a large right frontal tumor with the surrounding edema. The trainees were prompted to consider treatment for symptomatic cerebral edema.

#### Case 3

In case 3, the patient was comatose. Given the inability to intubate a live actor and the discomfort of coma examination, here, both groups interacted with an intubated manikin. At the start of the case, trainees needed to recognize hypertension and bradycardia. Trainees were required to perform a focused coma examination and request appropriate labs and imaging. Once obtained, CT imaging demonstrated a large pontine hemorrhage with resultant hydrocephalus. Trainees were then required to identify that the treatment of hypertension, reversal of anticoagulation, and consultation to neurosurgery were the appropriate next steps.

### Learner Knowledge and Confidence Assessment

Medical knowledge about the management and workup of neurologic emergencies was assessed before and after the course using a 40-question multiple choice test developed by the course facilitators for the sole purpose of this course (C.A. and S.F.Z.). These questions were based on the American Heart Association/American Stroke Association (AHA/ASA) Acute Ischemic Stroke Guidelines,^13^ the AHA/ASA Guideline for Intracranial Hemorrhage,^14^ and the Neurocritical Care Society Guidelines for the Evaluation and Management of Status Epilepticus.^15^ An analysis of the theme of each question is available in the supplementary material (links.lww.com/NE9/A3). Participants were not told about the content areas before the test. The test was distributed by email 1 week prior to the course, and trainees were instructed to answer questions to the best of their ability without using any outside resources. They did not receive any explanations or receive a score after the pretest. The posttest questions were distributed by email immediately after the course, and participants were asked to complete it within 3 days. After the completion of all course work, residents received an answer key with detailed explanations for all questions.

Trainees were also surveyed on their confidence in managing various neurologic emergencies before and after the simulation. To create internal validity, we surveyed 9 neurologic emergencies that were included in the course learning objectives and 6 that were not (eTable 3, links.lww.com/NE9/A3). Trainees were asked to rate their confidence on a scale of 0–4, where 0 = not at all confident, 1 = not very confident, 2 = somewhat confident, 3 = very confident, and 4 = extremely confident. After the course, trainees completed the same confidence survey.

A component of the anonymous course feedback included directly eliciting trainee sentiment on the effect of an SP on the realness of the scenario and quantity of learning. Trainees in the standardized patient group were asked if they felt that SPs increased the case realness and, separately, if the SPs increased the amount they learned. Their options were to select yes or no accordingly. The trainees in the manikin-only platform were asked to consider if they thought SPs would improve the course as well as if they thought that SPs would have increased the amount they learned. All trainees were asked to rate the session for enjoyment and applicability.

Some residents (n = 5) had already had a 2-week neurologic intensive care unit (NeuroICU) rotation before their simulation course. In an effort to account for any confounding effect of NeuroICU time before the course, trainees were asked to select yes or no regarding prior NeuroICU time on the precourse survey and knowledge test.

### Behavior Checklist and Debriefing

To guide and facilitate directed debriefing, a detailed behavior checklist (eTable 1, links.lww.com/NE9/A3) was used to record what trainees did or failed to do during the simulation. Points on the checklists were derived from society-endorsed protocols for the management of each condition.^13-15^ The facilitator (C.A.) completed the checklists during each case. A short PowerPoint presentation was given at the end of each debriefing session which covered high-yield material and ensured all groups received the same core teaching.

### Equipment and Personnel

The SimMan Essential manikin (Laerdal Medical, Wappingers Falls, NY) was used in our cases. The required neurologic examination findings were portrayed on request using video supplements of real neurologic findings. These were obtained from consented patients who agreed to the use of their examination for teaching purposes. These were trimmed into individual examination components—for example, 1 clip demonstrated a patient's pupillary response to light. For the pontine hemorrhage coma case, the manikin was intubated prior to resident engagement. When requested by trainees, the monitor displayed heart rate and rhythm, blood pressure, and oxygen saturation. SPs were provided the script for the stroke and seizure case and attended a separate training date where they received teaching by a neurologist in how best to demonstrate neurologic examination findings including seizure-like activity and stroke.

### Statistical Analysis

Statistical analysis was performed with SPSS version 24. A 2 × 2 repeated measures analysis of variance was used to assess for differences from pretest to posttest for the 2 groups. Partial eta squared was used to calculate the effect size. An independent *t* test was used to test for differences between the 2 independent groups, such as those with NeuroICU experience and those without. Chi square was used to evaluate differences in proportions of residents on relevant measures. Statistical significance was *p* = 0.05. Effect sizes are reported for each analysis.

### Data Availability

The deidentified participant data for all surveys and testing are available by request. The details of each case are included in the supplemental material (links.lww.com/NE9/A0, links.lww.com/NE9/A1, links.lww.com/NE9/A2). The surveys, the knowledge test, and the teaching PowerPoints are available by request.

## Results

Baseline demographics of the participants are described in Table 2.

**Table 2:** Baseline Demographics of Participants

	Standardized patient group (N = 10)	Manikin group (N = 10)
Average weeks of neurology experience at the time of course	2.8	3.5
% Male	50 (n = 5)	30 (n = 3)
Had completed a NeuroICU rotation, n (%)	2 (20)	3 (30)
Average number of weeks of ICU time as an intern	1.9 (range 1–4)	2.4 (range 1–4)
Average number of neurology weeks during intern year	1 (range 1)	1.2 (range 1–2)
% Who cared for patients with stroke as interns	10 (n = 1)	40 (n = 4)
% That had calculated an NIHSS before the course	20 (n = 2)	40 (n = 4)
% That had been a responding clinician for a seizing patient before the course	50 (n = 5)	60 (n = 6)

Abbreviations: NeuroICU = neurologic intensive care unit; NIHSS = NIH Stroke Scale.

### Knowledge

**Figure 1:**
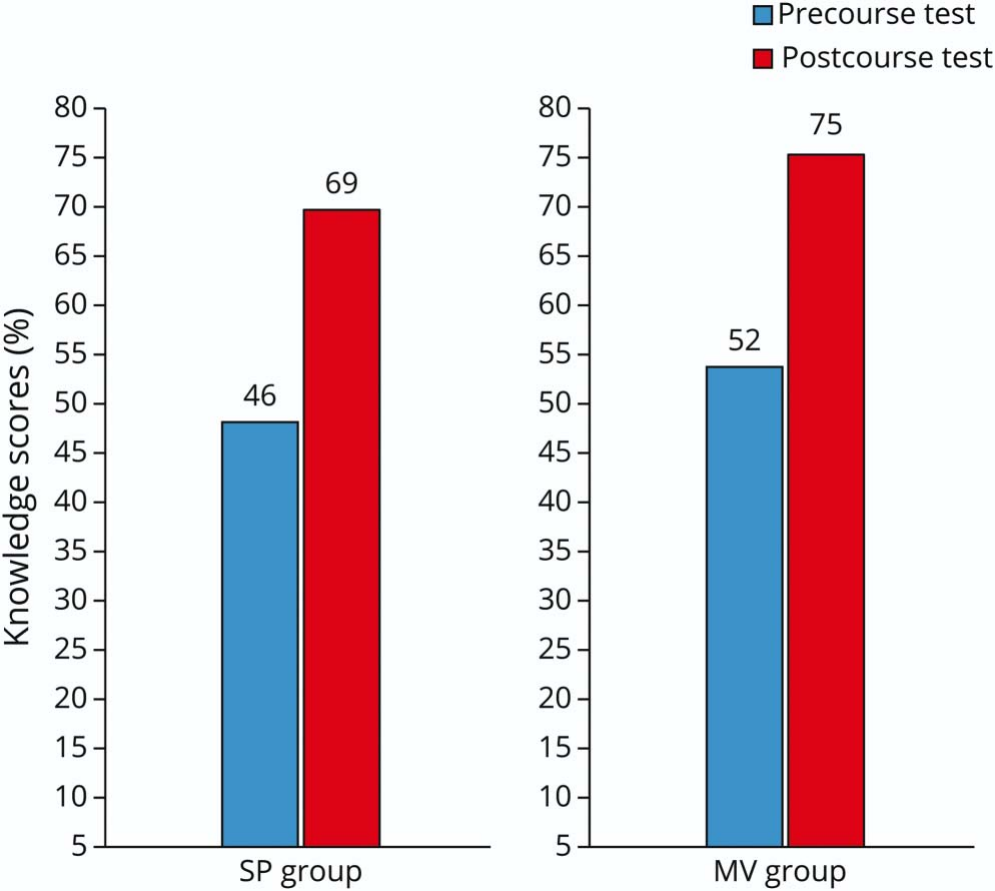
Increase in Knowledge Scores Diagram demonstrating an average trainee score on the 40-question knowledge quiz both before (blue) and after (red) the course. Scores are reported by group assignment.

Figure 1 displays the average score on the 40-question multiple-choice knowledge test, which was administered before and after the course. Thematic analysis of the questions is available in the supplemental material including an analysis of percent correct on each question before and after the course (eTable 2, links.lww.com/NE9/A3). Using analysis of variance, there was a significant improvement from baseline scores to postcourse scores (*p* < 0.001, effect size 91%). There was no significant difference in the acquisition of knowledge between the 2 groups; each group's score increased by 23% (*p* = 0.977). Precourse knowledge scores did not differ significantly as time went on (*p* = 0.905).

Among the 5 residents who had completed a 2-week NeuroICU rotation prior to simulation, the average pretest knowledge score was 49%, exactly the same as those residents who had not yet rotated in the NeuroICU (*p* = 0.939).

### Confidence

Confidence ratings were obtained from 19 of the 20 residents' preintervention and all 20 residents' postintervention.

#### Preintervention

**Figure 2:**
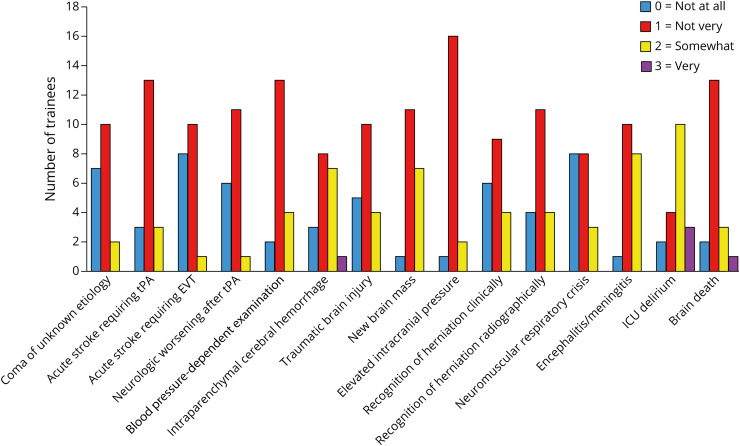
Baseline Confidence Scores Among All Trainees Diagram presenting the number of trainees who selected each confidence score by topic before the course. Total number of responses for each emergency (n = 19). No resident selected score 4 (extremely confident).

**Figure 3:**
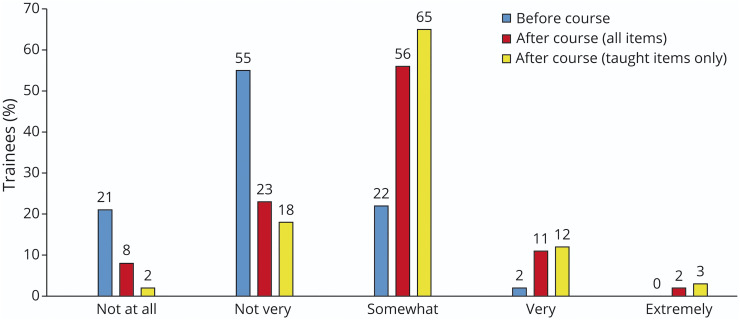
Shift in Self-Assess Confidence Before and After the Course Among All Trainees Frequency (by %) of each confidence score (0–4) over the 15 surveyed items reported by all trainees both before the course (blue) and after the course (red). Before the course, 19 residents scored 15 items for a total of 285 scores. For example, not at all confident (score 0) was reported 59 times (21% of the time). After the course, 20 residents scored 15 items for a total of 300 scores. For example, not at all confident (score 0) was reported 23 times (8% of the time). The yellow bars demonstrate the percentage of each confidence score when only the 9 items which were directly taught in the course were surveyed. Twenty residents scored 9 items for a total of 180 scores. For example, not at all confident (score 0) was reported 3 times (2% of the time).

Figure 2 shows baseline confidence scores by topic before the course. Figure 3 demonstrates the percentage of scores in total before and after the course. Before the course, “not at all confident” (score 0) represented 21% of responses. “Not very confident” (score 1) represented 55% of responses and was the median score for all topics except the management of ICU delirium. “Somewhat confident” (score 2) was selected in 22% of precourse responses and was the median score for the management of ICU delirium. “Very confident” (score 3) was selected very rarely (2% of responses) in the baseline assessment, and “extremely confident” (score 4) was never selected in the precourse assessment.

#### Postintervention

After the course, the percentage of all trainees selecting somewhat confident (score 2) and higher-level confidence scores increased (Figure 3). Not at all confident (score 0) was selected in 8% of postcourse responses; not very confident (score 1) in 23% of responses. Somewhat confident (score 2) increased to 56% of responses, and very confident (score 3) increased to 11% of postcourse responses. Extremely confident (score 4) which was never selected before the course increased to 2% of postcourse responses. When we examined only the 9 topics that were directly taught in the course, the percentage of higher confidence scores was more pronounced as depicted in the white bars of Figure 3 (score 0—2%, score 1—18%, score 2—65%, score 3—12%, and score 4—3%).

**Figure 4:**
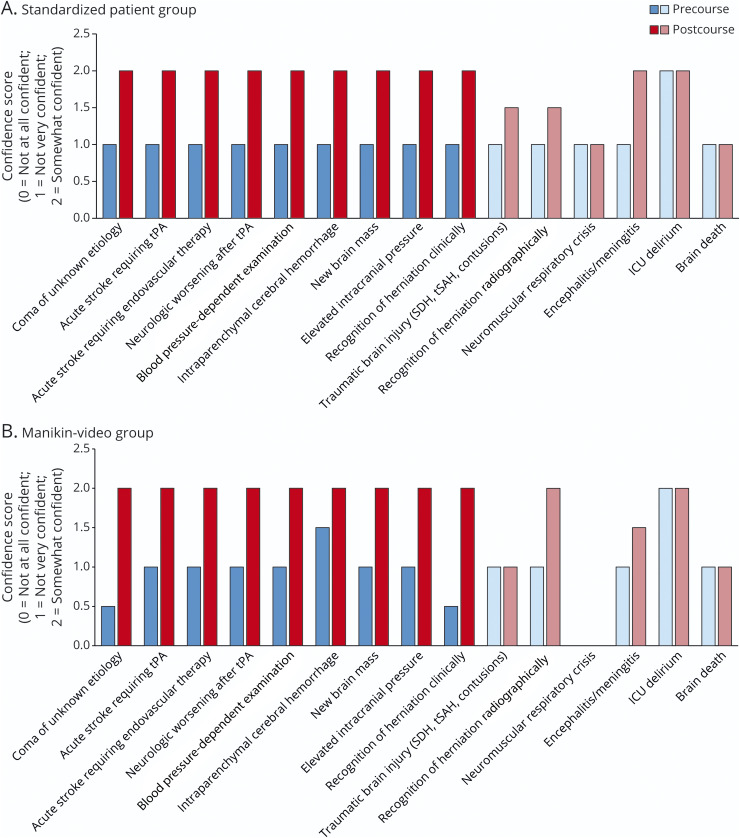
Median Confidence Scores Before and After the Course by Group Diagram demonstrating the change in median confidence scores by group assignment across all 15 items surveyed. Darker bars signify the topics that were taught in the course. Lighter bars represent the topics that were surveyed, but those were not addressed as part of the curriculum.

A comparison of precourse and postcourse median confidence scores by topic and by group assignment is shown in Figure 4. For all topics taught (darker bars), the median confidence score was 2 (somewhat confident) after the course, regardless of group assignment. While the median score postcourse was the same regardless of group assignment, score 3 (very confident) and score 4 (extremely confident) were more frequently reported in the SP group (17% vs 11%), but this difference did not reach statistical significance (*p* = 0.08).

#### Control Element

To provide internal validity, we surveyed residents on 15 neuroemergency topics, but only 9 were represented in the course curriculum. The median precourse score in 9 topics that were part of the course curriculum was 1, and as reported, the median score after the course for these topics was 2. The median precourse score on the 6 items that were not directly taught was also 1; however, unlike the taught topics, the median postcourse score in these topics was unchanged.

### Trends in Observed Behavior Checklist

The behavior checklist (eTable 1, links.lww.com/NE9/A3) provided important insights about situations in which trainees deviate from the treatment guidelines for neurologic emergencies.

In the stroke scenario, 4 groups did not request capillary glucose but did evaluate the glucose when routine labs were resulted. In 3 groups, there was significant debate about ordering a CT angiogram of the head and neck as part of the initial scan; the debate centered around not knowing the patients' glomerular filtration rate. It is of importance that all groups had some uncertainty about criteria and timing to engage the endovascular team for thrombectomy. When the patient clinically worsened after tPA, only 1 group halted the tPA infusion while the cause of neurologic worsening was being discerned.

In the seizure case, points of variation included the amount of lorazepam given initially and in total—no group discussed the strategy recommended for status epilepticus: 0.1 mg/kg lorazepam up to 4 mg times 2 doses.^15^ Two groups did not give an antiseizure medication after the seizure, and their patient had a second generalized tonic clonic seizure. At that point, the nurse prompted them to give “a longer acting medication.” All but one group gave levetiracetam; the other gave fosphenytoin. There was considerable variability in the dose administered.

In the case of pontine hemorrhage causing coma, all but one group quickly recognized the need to reverse anticoagulation. However, among the groups that did recognize the need to reverse anticoagulation, there was uncertainty about the dose of vitamin K and prothrombin complex concentrate to administer. Three groups recognized the need to urgently hold sedation to obtain a neurologic assessment; 2 groups opted to proceed with urgent imaging without ever performing a physical examination. No group assessed for nuchal rigidity. Only one of the 5 groups initially requested the CT angiogram despite basilar thrombus being a potential etiology for the physical findings.

Common themes that emerged during debriefing included when to obtain vascular imaging in acute stroke, when to intubate or call for help for status epilepticus, choice and initial dose of antiseizure medications, and when and how to reverse anticoagulation.

### Trainee Feedback

All residents completed a postcourse anonymized survey about their simulation experience. Regardless of group assignment, all 20 residents rated the simulation favorably, perceived the course to be worth their time, and felt the course was applicable to their practice. All but one trainee felt the course lessened anxiety about practice in these high-stake scenarios.

When asked in the survey, 7 of trainees in the MV group felt that the use of standardized patients would have improved the course, but only 2 residents in this group felt that the use of standardized patients would have improved their knowledge gain. In the SP group, 8 of the trainees felt that the standardized patient increased the case realness and 8 also felt that the standardized patient increased the amount they learned.

## Discussion

Our study suggests that novice trainees gain both confidence and medical knowledge in the management of neurocritical care concepts through a simulation course, regardless of whether a standardized patient or manikin-video platform is used. Prior studies in neurology have used manikin-based curricula,^4,6,7,9,10^ standardized patient-based curricula,^3^ or a hybrid of the 2,^5,8^ but this study provided a direct comparison between the 2 platforms.

Regardless of group assignment, trainees equally improved in their performance on the postcourse test and in their reported confidence in the management of neurologic emergencies. The improvement in confidence was more strongly evident in the 9 topics that were part of the curriculum than in the 6 control items, which provides an element of internal validity for our findings. Our findings suggest that neurology residents benefit from participating in deliberate practice of managing neurologic emergencies. Similar to what has been demonstrated in the trauma literature, simulation training modality appears to have little effect on the perceived and actual education outcomes.^18^ This preliminary finding may support educators in selecting the simulation format that is most cost-effective for their program. For our study, the manikin-platform's only cost was the small honoraria provided to the senior residents who helped run the manikin. Conversely, training and participation of the standardized patients totalled over 1,000 dollars in cost. However, we recognize that other programs' expenses may vary.

Despite a lack in objective difference in the examination score or self-reported confidence measures, 8 of the 10 trainees in the SP group answered “yes” when asked in the anonymous feedback form “did working with a standardized patient increase the amount you learned?” This may be due to the ability to practice eliciting certain examination findings when the simulated encounter includes an SP, or it may be because showing video-tapped examinations resulted in frequent pauses and may thus disrupt trainee belief in the realism of simulation. Another potential benefit of standardized patients is their ability to provide communication and more direct feedback to the participants. Although this was not specifically assessed in our study, further research is needed to determine how standardized patients may improve the simulation experience or result in educational benefits that were unmeasured in this study.

It is interesting that we found that the average pretest score for residents who had rotated in the NeuroICU prior to the course was the same as that of those with no NeuroICU exposure. This occurred despite 3 of the residents being currently in the NeuroICU on the day they took the pretest, and the other 2 within 1 week of finishing a block of NeuroICU time. This may be because all residents were very early in their PGY-2 year and thus had an intrinsically high cognitive load in adapting to their new positions. It may also reflect trainees' different exposure to neurologic emergencies in internship or medical school. However, of the NeuroICU-experienced residents, those who had completed night rotations had higher pretest scores than their peers. We hypothesize that this may result from greater autonomy at night and the higher probability of being the first responder to an emergency. Although not powered to detect a statistical difference, this finding suggests that trainees would benefit from more intensive teaching methods and that traditional bedside teaching may be inadequate in reinforcing critical care concepts for novice learners.

The scores on the precourse test (eTable 2, links.lww.com/NE9/A3) and areas of performance variation based on the behavior checklist (eTable 1) identified important areas to address in future educational programming. For example, from the pretest items regarding status epilepticus, we determined that only 10% of trainees initially identified the correct dosing for second-line agents in status epilepticus and only 20% recognized bradycardia and hypotension as contraindications for giving fosphenytoin.^18^ In the simulations, there was uncertainty about the timing for administration of antiseizure medications. Such misunderstandings reflect previously observed clinical treatment deviations. In status epilepticus, for example, it has been shown that nearly 40% of the patients do not receive guideline-recommended management.^19^ Such deviations by experienced clinicians may be due to necessary dosage adjustments aimed at tailoring treatment to the individual, but these personalized decisions may confuse novice trainees who do not yet have the experience in making such decisions.

Regarding ischemic stroke management, pretest questions involving the performance of the NIHSS revealed that many novice trainees do not correctly score hemianopsia, neglect, ataxia, and dysarthria (eTable 3, links.lww.com/NE9/A3). During the acute ischemic stroke case, trainees frequently debated when to order vascular imaging and when to consult the endovascular team. Pinpointing these critical learning points is essential in ischemic stroke because every 30-minute increase in computed tomography-to-reperfusion time has been shown to reduce the probability of achieving a functionally independent outcome by 8.3%.^20^ Indeed, for acute ischemic stroke, simulated practice has already been shown to have a measurable effect at the bedside: resulting in a swifter door to needle time in tPA administration.^3,4^

In assessing knowledge on pontine hemorrhage causing coma, only 30% of trainees correctly answered a question on how long etomidate and succinylcholine would affect the ability to obtain a reliable neurologic examination. The differential for coma of unclear etiology includes basilar thrombosis. Most groups did not discuss this possibility. While is it plausible that basilar thrombosis would have been entertained had the pontine hemorrhage not been identified, our finding in simulation mirrors the real-world delays in the diagnosis of vascular causes of coma^21^ and, importantly, offers a chance to provide corrective instruction prior to patient encounters.

Regarding resident satisfaction, all residents evaluated the course favorably. Despite the loss of a free morning, all residents reported that the training was “worth the time it took.” We hypothesize that this high level of engagement and satisfaction resulted from direct applicability of the course and it being scheduled directly prior to critical care or emergency department shifts, although further studies would be needed to confirm this.

Limitations of the study include that it is a single-center pilot. That no statistical difference was found between the 2 platforms is likely because our study was underpowered. Using our existing effect size for differences between the 2 simulation formats for knowledge gain on the written test, a future study with at least 84 trainees in each arm would be required to detect a statistically significant difference with a power of 0.80 and an effect size of 0.10. The use of the manikin for both groups for the third case would also have diluted our ability to detect differential effects in our overall analysis. In addition, we assessed residents working as groups. The experience might be different if 1 resident were working on their own or if the simulation was part of a summative milestone assessment and not just a formative experience. This would also allow stronger comparison between residents who had completed ICU time vs those who had not. Although their knowledge scores were the same in this study, important differences might be detected in their performance of the simulations.

There are limitations to written testing as an assessment strategy. Because learners took the same precourse and postcourse test, some of the improvement on the test may have been due to test-taking learning. Long-term follow-up and in situ evaluation could offer more meaningful evaluation of resident performance in a neurologic emergency and should be considered for future studies.

Finally, there are limitations in basing the task checklist on society guidelines for single diagnoses. Many neurocritically ill patients have competing high-priority needs. For example, in ICH, there is an urgent need to obtain imaging. However, ICP crisis resulting from ICH also needs to be emergently managed, and this ICP crisis may worsen when laying a patient flat to obtain the necessary imaging. In our case, despite clear concern of herniation, trainees were not expected to administer hyperosmolar therapy until they saw evidence of herniation on the CT scan because the management of herniation is addressed in the guidelines downstream of obtaining neuroimaging. These limitations can be addressed by widening the scope of each clinical scenario; thus, helping trainees obtain the critical thinking and triage decision skills that are paramount in the management of these complex patients.

Our study suggests that trainee knowledge and confidence in the management of neurologic emergencies increase after a simulation experience, irrespective of the specific simulation platform used. Future larger studies are needed to assess for a differential increase in knowledge between live simulation vs manikin use. The use of checklists and a nonjudgmental debriefing approach uncovered important variations in protocol adherence among novice physicians, as well as the areas of management uncertainty. Given these findings, we recommend that all neurology programs consider modern approaches to simulation and supportive debriefing as one method to identify and address learning needs in the management of neurologic emergencies for junior residents.

## Appendix Authors

**Table:**

Name	Location	Contribution
Catherine S.W. Albin, MD	Department of Neurology, Emory University School of Medicine, Atlanta, GA	Drafting/revision of the manuscript for content, including medical writing for content; major role in the acquisition of data; study concept or design; analysis or interpretation of data
Emil Petrusa, PhD	Department of Surgery, Massachusetts General Hospital, Harvard Medical School, Boston	Drafting/revision of the manuscript for content, including medical writing for content; study concept or design; analysis or interpretation of data
James A. Gordon, MD	Department of Emergency Medicine, Massachusetts General Hospital, Harvard Medical School, Boston	Drafting/revision of the manuscript for content, including medical writing for content; study concept or design; analysis or interpretation of data
Deepa Malaiyandi, MD	Department of Neurology, University of Toledo, OH	Drafting/revision of the manuscript for content, including medical writing for content
Sahar F. Zafar, MD	Department of Neurology, Massachusetts General Hospital, Harvard Medical School, Boston	Drafting/revision of the manuscript for content, including medical writing for content; major role in the acquisition of data; study concept or design; analysis or interpretation of data

## Glossary

AHA: American Heart Association
ASA: American Stroke Association
group MV: manikin with video supplement group
group SP: standardized patient group
ICP: intracranial pressure
MCA: middle cerebral artery
NeuroICU: neurologic intensive care unit
NIHSS: NIH Stroke Scale
PGY: postgraduate year
tPA: tissue plasminogen activator
